# Nature’s Uniformity

**Published:** 1875-01

**Authors:** 


### NATURE'S UNIFORMITY.

Nature's laws, as well as the accidents and incidents of life, are very uniform ; it is thus that life insurance companies are able to make their calculations with pecuniary safety. Of fifty men in good health at fifty years, a certain number on an average will live twenty-five years longer, hence by insuring all of them to live ten years longer, they are certain to make money, as they always charge a profit. The average heat or cold, or rain-fall of one year, is generally equal to that of another. The number of suicides, or accidental deaths in any great city, is generally proportioned to the number of inhabitants. The expenses of our government since its foundation, show a curious uniformity, leaving out the accident of war. The expenses of the government during Washington's administration, in proportion to the number of the people, was a tax on each person equal to one dollar and thirty cents. In General Grant's, leaving out the war, it is one dollar and seventy cents, varying but five cents from the earlier administrations of Adams and Madison, while it is even below those of Monroe, Polk, Pierce and Buchanan. But in the war of Lincoln's administration, the cost of government was seventeen dollars for each man, woman and child. The death rate of large cities is about the same from year to year ; twenty-two or twenty-three out of every thousand for years together in London, and for every man that dies, twenty-eight persons have been sick one day.

